# Regulatory role of PPAR in colorectal cancer

**DOI:** 10.1038/s41420-025-02313-2

**Published:** 2025-01-28

**Authors:** Cong Wang, Tingcong Lv, Binghui Jin, Yang Li, Zhe Fan

**Affiliations:** 1 Department of General Surgery, The Third People’s Hospital of Dalian, Dalian Medical University, Dalian, China; 2 Department of General Surgery, The Third People’s Hospital of Dalian, Faculty of Medicine, Dalian University of Technology, Dalian, China; 3 Department of Breast Surgery, Cancer Hospital of China Medical University, Shenyang, China; 4 Department of Breast Surgery, Liaoning Cancer Hospital & Institute, Shenyang, China

**Keywords:** Colorectal cancer, Colorectal cancer

## Abstract

Colorectal cancer (CRC) is one of the most common tumors in the digestive system, and the majority of patients are found to be in advanced stages, which is a burden to human health all over the world. Moreover, in recent years, CRC has been progressively becoming younger, with an increasing incidence mainly among patients <50 years old. Despite the increase in awareness of CRC and the continuous improvement of medical treatment nowadays, the challenge of CRC still needs to be conquered. By now, the pathogenesis of CRC is complex and not fully understood. With the deepening of research, it has been revealed that PPARs, as a transcription factor, are inextricably linked to CRC. This article outlines the mechanisms by which PPARs are involved in CRC development. An in-depth understanding of the pathways related to PPARs may provide new ways of developing effective therapies for CRC with PPARs as potential targets.

## Facts

- PPAR is a nuclear receptor that exists in three subtypes: PPARα, PPARβ /δ, and PPARγ. They function as transcription factors and can participate in tumorigenesis, cell proliferation, angiogenesis, cell death, invasion, migration, and tumor metabolic processes.
- Most current studies have shown that PPARα and PPARβ /δ are highly expressed, while PPARγ is suppressed in CRC.
- Although the role of PPARs in CRC is not fully understood, developing novel therapies targeting PPARs has become a new trend.

## Open questions

- What are all the mechanisms by which PPARs affect CRC progression through the tumor microenvironment?
- Is targeted therapy for PPAR universal, and does this need to be validated by more clinical results?
- Can PPAR expression levels be used as a screening and prognostic indicator for CRC and indicate patient condition?
- Can PPAR modulators be used in combination with each other and with other therapies, and is there consistency in the role of their results in CRC?

## Background

Colorectal cancer (CRC) represents the second most common cause of mortality worldwide. Many physiological and pathological mechanisms, including cell proliferation, angiogenesis, cell death, invasion and metastasis, and tumor metabolism, regulate the development and progression of CRC [1]. In recent years, researchers have made progress in CRC through continuous research. For example, they have identified many new diagnostic biomarkers and therapeutic targets that positively affect the diagnosis and treatment of CRC patients [2–5]. However, the incidence of CRC in patients <50 years of age has risen globally over the past decade. CRC has become the third leading cause of cancer deaths in people under 50 years of age. This rising trend may be related to risk factors [6, 7]. Common risk factors for CRC include genetic susceptibility, diet, obesity, alcohol consumption, infections, and lifestyle changes [1, 8–10]. Despite significant advancements in the diagnosis and surgical treatment of CRC, the occurrence of metastasis and recurrence continues to reduce patient survival rates [11, 12]. These reasons urge us to conduct further in-depth studies on CRC’s relevant transcriptional modes and pathways. This will facilitate the identification of efficacious targets for the prevention and treatment of cancer.

### Transcription of PPAR

PPAR is an endogenous or exogenous ligand-activated transcription factor belonging to the nuclear hormone receptor superfamily. It currently exists in mammals in the form of three subtypes, including PPARα, PPARβ/δ, and PPARγ [13]. Binding of PPARs to ligands prompts binding to co-repressor proteins and heterodimerization with the retinoic acid X receptor. During this process, the protein conformation is altered, releasing co-repressors (e.g., SMRT and NCoR) and recruiting co-activators (e.g., p300, SRC-1). This process involves many transcriptional co-activator interactions, including PPARγ co-activator-1 (PGC-1) α and PGC-1β. The binding of this complex to the PPRE consensus DNA sequence (AGGTCANAGGTCA) in the promoter of the target gene, thereby regulating the transcription of the target gene. It regulates gene expression and cellular functions and is distributed in different tissues to perform biological functions [14–17].

### Classification and physiological significance of PPAR

PPARα is the first subtype to be discovered [18]. It primarily regulates lipid and glucose dynamic homeostasis, the mobilization and catabolism of fatty acids, and the enhancement of fatty acid oxidation (FAO). PPARα is highly expressed in the liver, kidney, intestine, heart, skeletal muscle, and brown adipose tissue [18–20]. PPARβ/δ is widely distributed in various tissues, with the highest expression levels observed in colonic epithelial cells, skin, and adipocytes. The primary functions of PPARβ/δ are the regulation of glucose and lipid homeostasis, cellular energy expenditure, and blood lipid levels. Additionally, it stimulates lipogenesis, facilitates wound healing, and enhances resistance to adverse environmental conditions [19–22]. PPARγ has been extensively studied, and PPARγ is expressed in kidney and intestinal mucosa and white and brown adipose tissue. It is a primary regulator of adipocyte generation and differentiation and a key regulatory factor of lipid metabolism [17]. In addition to this, it is implicated in developmental processes. Given the involvement of different promoters in the coding process, PPARγ gives rise to two main subtypes: PPARγ1 and PPARγ2. PPARγ1 is expressed in numerous tissues, whereas PPARγ2 is solely expressed in adipose tissues but can be induced by high-fat diets (HFDs) in other tissues [21]. PPARγ is more inclined to exert favorable human anticancer effects [23].

PPAR plays a crucial role in regulating lipid metabolism, glucose metabolism, and the control of energy homeostasis. Moreover, it is also involved in other biological processes, including cell proliferation, differentiation, apoptosis, and angiogenesis [24, 25]. PPAR has been implicated in a wide range of human diseases. The role in developing certain diseases such as atherosclerosis, inflammation, immunity, cancer, and various metabolic disorders like obesity and diabetes mellitus is well-established [19, 24].

### PPAR in CRC

Dysregulation of lipid metabolism is one of the important pathways for cancer formation and progression, and it is a key factor in the acquisition of energy supply by cancer cells to regulate the tumor environment [26]. One of the most critical functions of PPAR is maintaining lipid metabolic homeostasis and energy levels. Therefore, the PPAR signaling pathway is involved in the regulation of the complex network of cancer and has an intricate relationship with tumors. PPAR plays a noteworthy role in controlling cancer growth [27]. In CRC, with tumor progression, increased expression of PPARα and PPARβ/δ or decreased expression of PPARγ [24]. Thus, PPAR may have potential pharmacological significance in colon cancer [28]. Nevertheless, the function of PPAR in CRC remains incompletely elucidated. PPAR may serve as a tumor suppressor or a tumor-promoting factor, exerting antitumor or pro-tumor effects, respectively. The role of PPAR in CRC remains inconclusive due to the inconsistency of results observed across different animal models and human tissues. This variability is likely attributed to the influence of internal and external environmental factors [18, 29].

Cancer has a complex and precise ecosystem network containing tumor and non-tumor components. The interactions and crosstalk mechanisms between the two promote the development and progression of solid tumors [30, 31]. Cancer is characterized by unlimited cell proliferation and cell cycle imbalance [32]. The development of tumors is not a single process involving complex multi-mechanistic pathways. The operation of these pathways and mechanisms allows PPARs to play an important role in “cancer hallmarks [33].” This article will describe the mechanism and role of PPARs in CRC in several ways using the framework of the “cancer hallmarks” defined by Hanahan and Weinberg.

## Mechanism and role of PPARα in CRC

### Tumorigenesis and proliferation

The current study suggests that PPARα activation has an antiproliferative effect [34, 35]. It has been shown that PPARα deficiency enhances tumorigenicity in mice by mediating the RB1/E2F pathway to increase DNA methyltransferase 1 (DNMT1)-mediated p21 methylation and protein arginine methyltransferase 6 (PRMT6)-mediated p27 methylation. When the PPARα agonist fenofibrate was administered, this was reversed and inhibited colon carcinogenesis by suppressing intestinal cell proliferation in mice [34]. Notably, the PPAR pan-agonist bezafibrate increased the proliferation and survival of tumor-reactive CD8^+^ T cells; it also enhanced the inhibition effect of colon cancer development in mice when combined with PD-1 blockade [36]. The data presented clearly indicate that PPARα plays a crucial role in the occurrence and development of CRC. It can be reasonably deduced that PPARα agonists may be promising drugs for treating CRC [34]. In contrast, a study demonstrated that PPARα antagonists exhibited antiproliferative effects on paraganglioma, pancreatic, and CRC cells. This indicates that PPARα antagonists may possess anticancer potential [37].

A recent study has shown that the function and characterization of cancer stem cells (CSCs), which are the leading cause of cancer recurrence and drug resistance, mainly depends on methionine. SIRT1/PGC-1α/PPAR-α regulation during methionine deprivation is involved in the impaired stemness of cancer cells, decreasing the self-renewal and differentiation potential of CSCs. Consequently, this mechanism provides a foundation for regenerative medicine and the treatment of cancer recurrence [38]. The regulation of CSCs represents a crucial aspect of tumorigenesis. Moreover, an imbalance in CSCs is associated with malignant tumors’ advancement and developmental potential. The PPAR has been identified as a pivotal factor in the growth of CSCs, as evidenced by numerous studies [39].

### Angiogenesis

Early studies have shown that PPARα agonists reduce transcriptional activation of COX-2 and VEGF, which are associated with tumor angiogenesis [40]. It has been described that the anti-angiogenic effect of pomegranate peel extract (PPE) is partly attributed to the activation of PPARα and PPARγ in human umbilical vein endothelial cells. Moreover, their antagonists can counteract PPE’s positive tumor angiogenesis inhibitory effects [41]. Although the anti-angiogenic effect of PPARα is well documented, some studies have demonstrated that it can also promote tumor angiogenesis [25, 42, 43]. This discrepancy based on the effects of different PPARα agonists in different animal models urges us to investigate further the mechanisms and outcomes of PPARα affecting angiogenesis in CRC.

### Cell death

Early experiments have indicated that the activation of PPARα plays a role in the apoptotic process in colon cancer [44]. Experiments conducted by Gao et al. using human SW480, HCT-116, and other cells suggested that PPARα, an E3 ligase, induces Bcl2 ubiquitination and degradation, leading to apoptosis [45]. Mesenchymal stem cells-derived extracellular vesicles harboring miR-378a-3p have been reported to mediate the GATA2/AQP4/PPAR-α signaling pathway. It inhibits apoptosis and inflammatory bowel disease (IBD) in the mouse colonic epithelial cell line M064 through PPARα inactivation [46]. There is growing evidence that CRC is one of the most severe consequences of IBD and that IBD is involved in the development of CRC [47, 48]. The down-regulation of carnitine palmitoyltransferase-1A (CPT1A), a pivotal enzyme in FAO, reduces intestinal inflammation, oxidative stress, and apoptosis through inhibition of PPARα expression in dextran sulfate sodium (DSS)-induced HT-29 cells. This result suggests that PPARα plays an essential protective role in ulcerative colitis [49]. It can, therefore, be posited that PPARα may play a role in regulating the development of CRC by influencing the process of IBD.

### Invasion and metastasis

N-acylethanolamine acid amidase inhibitor AM9053 inhibits tumor cell growth, proliferation, and migration via PPARα and TRPV1 in CRC cells. However, this antitumor effect can be disrupted by PPAR-α and TRPV1 antagonists [50]. Studies have shown that fenofibrate, as a PPARα agonist, is believed to reduce the methylation of the tumor suppressor gene CDKN2A by inhibiting the content and activity of DNMT1. Then, it regulates the cell cycle, promoting apoptosis and inhibiting cell proliferation via the RB/E2F pathway. Fenofibrate also reduces cancer cells’ invasion and migration ability [35]. In contrast, unlike the above findings, one mechanism promoting metastasis in ovarian cancer is the activation of the PI3K/Akt/NF-κB pathway by low doses of mono(2-ethylhexyl) phthalate in a PPARα-dependent manner. In a mouse model, the PPARα inhibitor GW6471 inhibited this metastatic effect [51]. These findings indicate a need for further exploration of CRC invasion and migration.

### Tumor metabolism

**Fig. 1:**
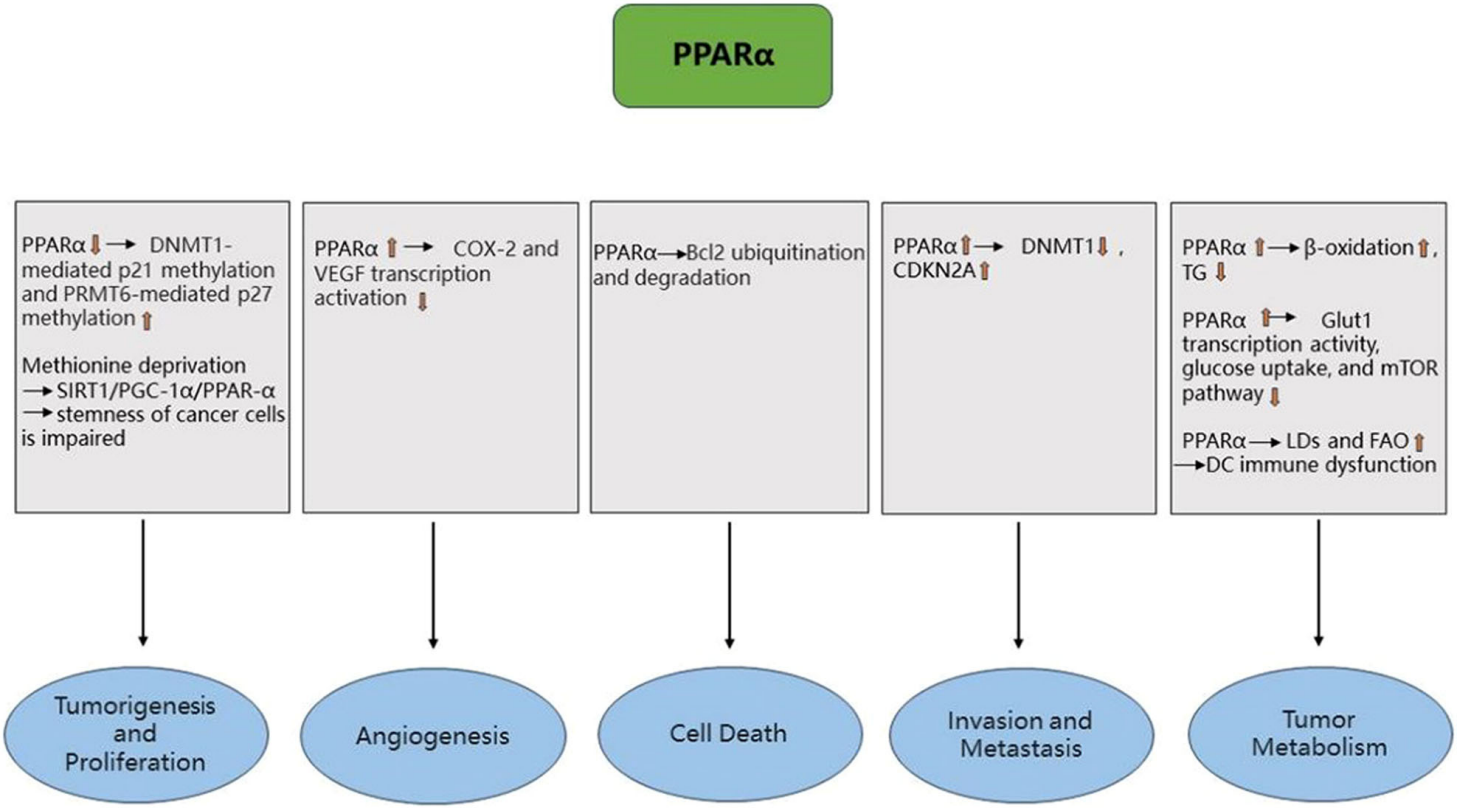
The role of PPARα in CRC. ↓ indicates inhibition and ↑ indicates increase.

Fibrate drugs, which belong to the class of PPARα agonists, can inhibit triglyceride synthesis by promoting β-oxidation, thereby modulating widely altered lipid signaling in cancer cells and impacting colon cancer survival and proliferation [52]. PPARα agonist Wy14,643 reduces CRC cell growth through inhibitory effects on Glut1 transcriptional activity, glucose uptake, and mTOR pathway [53]. Interestingly, a study demonstrated that increased bile acids lead to impaired Lgr5^+^ intestinal stem cell (ISC) function by inhibiting PPARα-mediated FAO. This may exacerbate the progression of colitis and promote colitis-associated colon cancer [54]. Nevertheless, some evidence suggests that PPARα may also exert a pro-cancer effect. Acid-adapted colon cancer cells have been observed to reorganize cancer cell metabolism by increasing PPARα activity and participate to some extent in cancer cell proliferation and invasion. Consequently, by exploiting the sensitivity of cancer cells to PPARα inhibition, PPARα inhibitors can impede this metabolic process for antitumor purposes [55]. Additionally, it has been shown that in a mouse colon cancer model, PPARα-deficient mice exhibit reduced tumor growth rates compared to wild-type mice. These PPARα-deficient mice improve the function of dendritic cells (DCs) by inhibiting PPARα, exerting a powerful antitumor effect. In other words, PPARα positively responded to the tumor-derived exosomes-mediated increase in lipid levels, which induced lipid droplets generation and FAO enhancement, resulting in DC immune dysfunction. Therefore, they suggested that inhibition of PPARα may also serve as one of the strategies for antitumor therapy [56]. However, more experimental findings are needed to support this. Figure 1 shows the role of PPARα in CRC.

## Mechanism and role of PPARβ/δ in CRC

### Tumorigenesis and proliferation

Less is known about PPARβ/δ than about the other two subtypes. The first evidence linking PPARβ/δ to cancer was found in CRC [57]. A study showed that elevated PPARβ/δ promotes colon tumorigenesis in an experimental mouse model [58]. Moreover, in subsequent studies, other investigators have demonstrated that colonic epithelial PPARβ/δ overexpression positively affects mice’s susceptibility to colon cancer [59]. They elucidated that PPARβ/δ overexpression in colon epithelial cells significantly enhances colon cancer susceptibility by promoting the expression of pro-tumorigenic genes, including IFITM3 and NRG1 [59]. In addition, Overexpression of PPARβ/δ also increases the incidence rate of colitis-associated colorectal cancer by mediating IL-6/activator of transcription 3 (STAT3) signaling to promote the expression of tumor-promoting genes, such as Notch3 and MUC1 [60]. However, some previous groups have obtained opposing results, with PPARβ/δ expression inhibiting CRC development and thus negatively regulating cancer progression [24, 61–63]. Along the same lines, other studies have shown that PPARβ/δ knockout mice have an increased risk of colon cancer carcinogenesis [64]. In the same way, the +294T/C (Rs2016520) SNP of *PPARβ/δ*, a downstream target gene of the Wnt/β-catenin signaling pathway, reduced the risk of CRC in a Mexican population [65]. The function of PPARβ/δ in the context of cancer remains a topic of contention within the scientific community. Given the inconclusive nature of the experimental results, using PPARβ/δ modulators in clinical practice cannot be recommended with certainty and should be cautiously undertaken.

### Angiogenesis

PPARβ/δ has a more pronounced pro-angiogenic function compared to PPARα and PPARγ [43]. A previous study showed that PPARβ/δ facilitates endothelial cell proliferation and angiogenesis by inducing the expression of the pro-angiogenic cytokine IL-8 through transcriptional and post-transcriptional mechanisms [66, 67]. Consistent with this description, subsequent studies have further demonstrated that the increase in IL-8 mediated by high PPARβ/δ expression promotes tumor angiogenesis and metastasis formation [68]. Findings on PPARβ/δ activation inducing endothelial cell proliferation and angiogenesis through a VEGF-dependent mechanism have been demonstrated [69]. Wagner and colleagues verified the effect of PPARβ/δ on angiogenesis. Using a vascular-specific overexpression PPARβ/δ transgenic mouse model, the authors concluded that PPARβ/δ promotes tumor angiogenesis, growth, and metastasis through activation of the PDGF/PDGFR, c-Kit, and VEGF/VEGFR pathways [70]. In subsequent experiments, they used RNA sequencing to demonstrate this conclusion [25]. In the HCT-116 CRC cell line and mouse colon tumor epithelial cells, GW501516 activated PPARβ/δ, which further induced an increase in the expression of COX-2 and its derivative PGE2 and led to an elevated expression of pro-inflammatory mediators in the colonic mucosa, including CXCL1, CCL2, CCL3, CCL4, and IL-1β. PPARβ/δ via this pathway is involved in tumorigenesis [63]. Interestingly, PGE2 also can promote colorectal tumor growth by transactivating PPARβ/δ [71]. Hence, there is positive feedback between PPARβ/δ, COX-2, and PGE2, which is a key factor in promoting tumorigenesis [72]. Furthermore, the expression of PPARβ/δ and COX-2 in CRC may promote angiogenesis and the risk of venous vessel invasion [73].

### Cell death

In 1999, He et al. initially demonstrated that adenomatous polyposis coli (APC) inhibits PPARβ/δ expression by interfering with the β-catenin/Tcf-4 pathway by analyzing whole gene expression profiles. Furthermore, alterations in this pathway, such as APC /β-catenin mutations, can increase PPARβ/δ activity. The nonsteroidal anti-inflammatory drug (NSAID) sulindac is thought to promote apoptosis in CRC cells by inhibiting PPARβ/δ expression [74]. Twenty years ago, the PPARβ/δ ligand GW501516 was validated in Apc(min) mice. This experiment established that activation of the anti-apoptotic pathway in intestinal epithelial cells by PPARβ/δ promotes intestinal tumor growth in mice [75]. Later, Liou and his colleagues found that PPARβ/δ can rescue apoptosis in CRC cells induced by inhibition of 14-3-3ε via NSAIDs such as sulindac. This further confirmed the role of PPARβ/δ in apoptosis [76]. The effect of NSAIDs on apoptosis always involves PPARβ/δ. 15-lipoxygenase-1 (15-LOX-1) expression is associated with NSAIDs, and its main product of metabolism, 13-S-hydroxyoctadecadienoic acid (13-S-HODE), promotes the apoptotic pathway by down-regulating PPARβ/δ expression [77]. A fascinating study found that high expression or activation of PPARβ/δ resisted the PPARγ-induced apoptosis effects in CRC cells. This resistance was mediated by survivin and caspase-3 [78].

### Invasion and metastasis

High expression of PPARβ/δ in colorectal, lung, and breast cancers affects metastasis-free survival of patients. This finding implies that PPARβ/δ regulates cancer metastasis [68]. Notably, when on an HFD, PPARβ/δ activation later directly binds to the Nanog promoter, increasing Nanog expression, which induces the amplification of CSCs and promotes hepatic metastasis of CRC. In contrast, the knockdown of PPARβ/δ attenuated this metastasis-promoting effect and inhibited tumor development. This finding is consistent with the pathway by which the PPARβ/δ agonist GW501516 promotes CRC metastasis [57]. Beyond this, AMPK-induced PPARδ-S50 phosphorylation inhibits colon cancer growth and metastasis by suppressing the transcriptional activity of PPARδ [79].

### Tumor metabolism

**Fig. 2:**
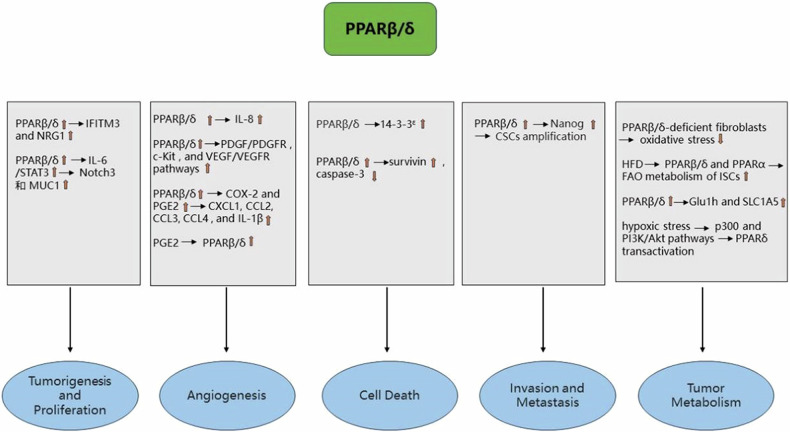
The role of PPARβ/δ in CRC. ↓ indicates inhibition and ↑ indicates increase.

It has been proven that the transcription factor MYC inversely regulates the expression of genes in the WNT signaling pathway. MYC promotes the interaction between LEF1 and β-catenin by directly targeting LEF1, activating the expression of PPARβ/δ and Acyl CoA dehydrogenase 9(ACAD9). This process ultimately results in metabolic reprogramming in colon cancer cells [80]. Impressively, fibroblasts deficient in PPARβ/δ in CRC cells regulate epithelial oxidative responses, reduce oxidative stress, and block the cell cycle. These mechanisms would reduce intestinal tumor load and decrease and delay CRC development [81]. Mana et al. showed that HFD activates FAO metabolism in ISCs via PPARβ/δ and PPARα, enhancing the tumorigenic potential. In addition, The PPARβ/δ agonist gw501516 has the ability to upregulate FAO-related genes [82]. Furthermore, HFDs and agonists increase the number of ISCs by upregulating PPARβ/δ levels, which is linked to the initiation and progression of CRC [83]. It has been proposed that the PPARβ/δ agonist GW501516 may contribute to the development of colitis-associated colon carcinogenesis, potentially through the increased expression of Glu1h and SLC1A5 [22]. This result is consistent with previous reports by these authors that PPARβ/δ promotes cancer cell proliferation and tumor progression through activation of Glut1 and SLC1A5 transcription [84]. These facts confirm that PPARβ/δ promotes metabolic signaling in tumors. In comparison, the AMPK agonist metformin inhibits GW501516-induced expression of Glut1 and SLC1A5 [85]. Besides, epidermal growth factor receptor (EGFR)-mediated phosphorylation of PPARδ-Y108 increased the stability of PPARβ/δ through heat shock protein 90(HSP90) and promoted the transcription of Glu1 and SLC1A5. This, in turn, regulates cancer cell proliferation and metabolism [86]. In HCT-116 cells, hypoxic stress induces transcriptional activation of PPARβ/δ. p300 and PI3K/Akt pathways may play a role in regulating PPARβ/δ transactivation under such hypoxic conditions. As a result‌, PPARβ/δ deficiency has been shown to inhibit the development of colon cancer in a hypoxic environment [87]. Figure 2 summarizes the role of PPARβ/δ in CRC.

## Mechanism and role of PPARγ in CRC

### Tumorigenesis and proliferation

Previous experiments have shown that PPARγ gene and protein expression is higher in rodent colon tumors than controls, suggesting a tumorigenic role for PPARγ [88]. Some studies have demonstrated that PPARγ activation increases the formation of colon polyps and promotes CRC [89, 90]. However, this view has changed. Most believe PPARγ activation can inhibit cancer development through various mechanisms [91]. Most of the findings indicate that PPARγ plays a significant place in inhibiting tumor proliferation, including colon cancer [9, 32, 91]. For example, the PPARγ agonist 15-d-PGJ2 suppresses tumor cytokine expression by inhibiting the NF-kB pathway in Caco-2 and HT-29 colon cancer cell lines. In addition, rosiglitazone, another agonist of PPARγ, was found to impede colorectal cell proliferation by activating PTEN (a tumor suppressor) and regulating the expression of phosphatidylinositol 3-kinase (PI3-kinase) [23]. On the other hand, HOXC11, an oncogene, may promote cell proliferation in colon cancer and renal clear cell carcinoma by down-regulating PPARγ signaling [92]. Promising evidence that PPARγ agonists prevent the survival of colon CSCs offers a new strategy for controlling cancer progression [32]. These findings open up potential possibilities for treating CRC with PPARγ agonists. The roles and mechanisms of the three PPAR agonists in CRC are shown in Table 1.

**Table 1:** Role and mechanisms of PPAR agonists in CRC.

PPAR agonists	Cancer cell lines	Mechanisms	Role in CRC	References
PPARα agonists				
Fenofibrate	–	P21 and P27 expression is increased, and P21 and P27 are negative regulators of the cell cycle	Inhibit	[34]
Fenofibrate	HCT-116 colorectal cancer cell line, SW480 and Caco-2 colon cancer cell lines, NCM460 and HIEC normal intestinal epithelial cell lines	Regulation of the CDKN2A/RB/E2F pathway through inhibition of DNMT1 content and activity to arrest the cell cycle in the G1 phase	Inhibit	[35]
Wy14, 643	SW480 and HT-29 colon cancer cell lines, HCT-116 colorectal cancer cell line	Inhibition of Glut1 gene transcriptional activity, glucose uptake, and mTOR pathway	Inhibit	[53]
LY171883 and WY14, 643	SW620 colorectal cancer cell line	Reduction of AP-1-mediated transcriptional activation of genes involved in the inflammatory response, such as COX-2 and VEGF, attenuates colon tumorigenesis at an early stage	Inhibit	[40]
PPARβ/δ agonists				
GW501516	HCT-116 colorectal cancer cell line	Increase IL-6/STAT3 signaling	promote	[60]
GW501516	HCT-116 colorectal cancer cell line, mouse colon tumor epithelial cells	Induces increased expression of COX-2 and its derivative PGE2, which increases the expression of pro-inflammatory mediators such as CXCL1, CCL2, CCL3, CCL4 and IL-1β	promote	[63]
GW501516	HCT-116 colorectal cancer cell line	Activation of anti-apoptotic pathways	promote	[75]
GW501516	–	Upregulation of genes associated with FAO	promote	[82]
GW501516	–	Increase number of ISCs	promote	[83]
GW501516	HCT-116 and LS-174T colorectal cancer cell lines	Lead to increased expression of Nanog, which induced the amplification of CSCs and promoted liver metastasis in CRC	promote	[57]
GW501516	SW480 colon cancer cell line, HCT-116 colorectal cancer cell line	Induction of Glu1h and SLC1A5 transcription	promote	[22, 84]
GW501516	HCT-116 and LS-174T colorectal cancer cell lines, HCA-7, HT-29, SW480 and SW620 colon cancer cell lines	Increase VEGF expression and activate PI3K-Akt signaling	promote	[77, 125]
PPARγ agonists				
(S)-3	HT-29 and RKO colon cancer cell lines	Upregulate p21^waf1/cip1^ and inhibit c-Myc and cyclin D1	Inhibit	[98]
15-d-PGJ2	Caco-2 and HT-29 colon cancer cell lines	Inhibition of the NF-kB pathway and suppression of cytokine expression in tumors	Inhibit	[23]
Rosiglitazone	CaCo-2 colon cancer cell line	Activates PTEN and blocks the PI3K/AKT signaling pathway, resulting in reduced cell proliferation	Inhibit	[23]
Rosiglitazone	CT26 colon cancer cell line	Remodel the tumor vasculature system, limit TAM infiltration, and inhibit the VEGF/VEGFR2 signaling pathway	Inhibit	[96]
Cladosporols A and B	HT-29 colon cancer cell line	inhibits adipogenesis in vitro	Inhibit	[17]
Thiazolidinedione	HT-29 Colon Cancer Cell Line	Induces cell differentiation and is associated with E-cadherin, β-catenin, Drg-1	Inhibit	[91, 115]
Thiazolidinedione	–	Inhibition of the activation of homodimeric and heterodimeric complexes of NF-κ B family members attenuates the immune response and reduces IL-8 expression	Inhibit	[126]
Troglitazone	HT-29 Colon Cancer Cell Line	Inhibition of MMP-7 synthesis and ECM protein adhesion	Inhibit	[42, 114]
Pioglitazone	HT-29 and SW480 colon cancer cell lines	Inhibition of COX-2 and cyclin D1 expression	Inhibit	[118]
13(S)HpODE	HCT-116 colorectal cancer cell line	Promotion of cancer cell apoptosis through IL-13 > 15-LO > 13(S)HpODE>PPARγ > MAO-A > ROS > P53 > p21 axis	Inhibit	[97]

### Angiogenesis

It is widely acknowledged that PPARγ activation facilitates the inhibition of tumor angiogenesis [43]. The tumor microenvironment (TME), which encompasses tumor cells and cancer‑associated fibroblasts, interstitial tissues, blood vessels, and a variety of inflammatory and immune cells, chemokines, and cytokines, is a complex and not yet fully understood system [93]. An excellent article confirms that overcoming TME can increase the sensitivity of Chimeric antigen receptor (CAR) T cells [94]. Subsequent reports have corroborated the assertion that heightened awareness of TME, the most significant obstacle, will enhance the efficacy of CAR T cell therapy in solid tumors [95]. It is worth noting that angiogenesis in TME is a crucial element influencing cancer progression. Based on mouse models constructed from the colon cancer cell line CT26 and breast cancer cell line 4T1, the PPARγ agonist rosiglitazone was found to remodel the tumor vasculature system and limit tumor-associated macrophage (TAM) infiltration. It also exerted anti-angiogenic effects in vitro by inhibiting the VEGF/VEGFR2 signaling pathway. The authors finally found that combining rosiglitazone with radiotherapy effectively inhibited angiogenesis, distant metastatic potential, and tumor recurrence [96].

### Cell death

It has been documented that the endogenous ligand 13(S)hydroperoxy octadecadienoic acid [13(S)HpODE] plays a role in colorectal tumor cell apoptosis through the MAO-A > ROS > P53 > p21 axis upon activation of PPARγ [97]. Chiral phenoxyacetic acid analog (S)-3 acts as a partial agonist of PPARγ, blocks the cell cycle, inhibits cell proliferation, and induces apoptosis through PPAR-dependent mechanisms: upregulation of p21^waf1/cip1^, and inhibition of c-Myc and cyclin D1 [98]. On the other hand, it has been reported that the PPARγ natural agonist, cladosporols, inhibits adipogenesis in vitro and suppresses tumor proliferation and invasive migration while promoting apoptosis. This mechanism of action provides a potential means of interfering with colon cancer growth [17]. In HT-29 human CRC cells, the PPARγ agonist rosiglitazone was observed to exert a protective effect in healthy intestinal tissues exposed to radiation, improve tissue structure, reduce intestinal apoptosis, and block inflammatory signaling cascades. These findings suggest that PPARγ agonists may effectively prevent and treat radiation-induced adverse conditions [99]. In addition, IFC-305, an adenosine derivative, inhibits methylation of the PPARγ promoter and up-regulates PPARγ expression, thereby reducing the risk of radiation-induced intestinal toxicity in colon cancer treatment [100]. It is well known that the Wnt/β-catenin signaling pathway plays an essential role in the development of CRC. β-catenin is a critical molecule in CRC [101]. Given that a potential crosstalk mechanism between PPARγ and Wnt/β-catenin regulates CRC progression, this offers a promising avenue for the development of novel PPARγ agonists that inhibit the Wnt/β-catenin signaling pathway [102]. Seetha and his colleagues found that the combination of indomethacin and juglone reduces the expression of inflammatory cytokines through the Wnt, Notch, and PPARγ pathways, thereby inducing apoptosis in colon cancer cells [103].

Non-coding RNAs (ncRNAs), which comprise the vast majority of the human genome, are a group of non-protein-coding RNAs [104, 105]. NcRNAs include microRNAs (miRNAs), long ncRNAs (lncRNAs), circular RNAs (circRNAs), and PIWI-interacting RNAs (piRNAs) [104–106]. Numerous studies have shown a potential association between ncRNAs and cancer development. NcRNAs modulate cancer by regulating gene expression and complex biological processes [105]. For example, circRNAs have been shown to regulate the progression of lung cancer, hepatocellular carcinoma, and glioma through the Wnt/β-catenin and PI3K/AKT pathways [104, 105, 107, 108]. MiRNAs can inhibit the PTEN/PI3K/Akt pathway, promoting growth in multiple myeloma (MM) [109]. Targeted miRNA-based therapies offer insights into potential treatments for glioma [110]. Of interest, a well-designed study by Zhang et al. using CRC cells showed that PPARγ is regulated by the LncRNA TINCR/microRNA-107/CD36 axis and that this pathway regulates tumor proliferation or apoptosis [111]. Surprisingly, a recent in vitro experiment concluded, contrary to the above, that inhibition of the PPARγ signaling pathway significantly inhibited the growth and accelerated the apoptosis of CRC cells [112]. A significant article summarizes the mechanisms through which pro-inflammatory cytokines, including the IL-1 family, IL-6, and TNF-α, exert pro-tumorigenic or tumor-suppressive effects during tumorigenesis. The levels of these cytokines within the TME may be correlated with cancer development [67]. A well-established example exemplifies that the PPARγ agonist 15-d-PGJ2 and troglitazone block the IL-6 signaling pathway through the inactivation of STAT3, which induces attenuated proliferation and increased apoptosis in MM cells [113].

### Invasion and metastasis

Previous reports have shown that the PPARγ agonist troglitazone inhibits colon cancer metastasis by inhibiting matrix metalloproteinase-7 (MMP-7) synthesis and the adhesion of extracellular matrix (ECM) proteins [42, 114]. Twenty years ago, as a result of the establishment of xenograft animal models, the PPARγ ligand thiazolidinedione was shown to have the ability to inhibit the growth and metastasis of colon cancer cells by promoting differentiation effects. These effects are strongly linked to E-cadherin, β-catenin, and Drg-1 [115]. Nevertheless, later, Papi et al. proposed that the combination of 6-OH-11-O-hydroxyphenantrene (IIF) with PPARγ ligands, including ciglitazone and pioglitazone, inhibited the proliferation and invasion of colon cancer cell lines [116]. A recent study demonstrated that the pro-carcinogenic effects induced by IL-33, a product of Group 2 innate lymphoid cells (ILC2s), are driven by PPARγ. This pro-carcinogenic effect is characterized by increased CRC’s invasive and metastatic capacity without any impact on cell proliferation. By contrast, inhibition of PPARγ interferes with this effect [117]. Furthermore, Takano and colleagues found that the PPARγ agonist pioglitazone down-regulated COX-2 and cyclin D1 and inhibited colon cancer proliferation and liver metastasis [118]. Apart from this, recent studies have shown that the utilization of pioglitazone does not prevent ischemia/reperfusion injury (IRI)-induced liver metastasis in a mouse colon cancer model of IRI [119]. At present, PPARγ exhibits a paradoxical role in metastasis. Given the lack of inadequate research efforts in this area, it is impossible to hypothesize the exact role of PPARγ in CRC metastasis.

### Tumor metabolism

**Fig. 3:**
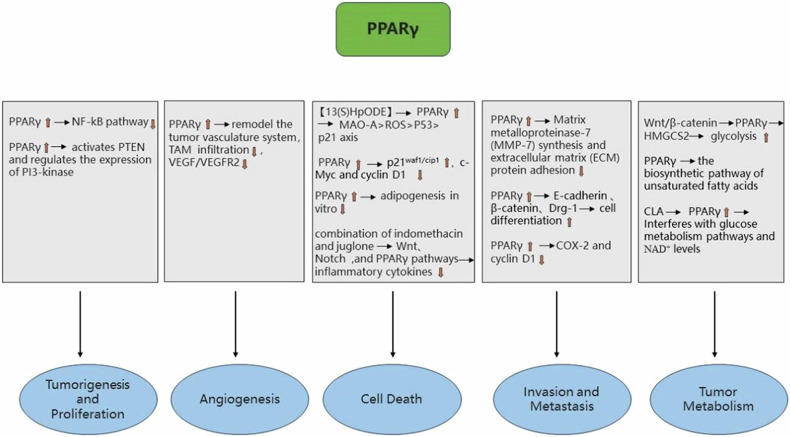
The role of PPARγ in CRC. ↓ indicates inhibition and ↑ indicates increase.

The Wnt/β-catenin pathway negatively regulates mitochondrial 3-hydroxy-3-methylglutaryl-CoA synthase 2 (HMGCS2) via PPARγ signaling. Subsequently, this results in an increase in glycolysis and the regulation of enterocyte differentiation [120]. Alternatively, the biosynthetic pathway of unsaturated fatty acids has been reported to contribute to the elevated risk of CRC observed in obese populations. Linoleic acid can inhibit this pathway, and PPARγ, which acts as a receptor for linoleic acid, plays an essential role in this process [121]. Further evidence substantiates the assertion that the upregulation of PPARγ by conjugated linoleic acid impedes colon cancer cell proliferation and stimulates apoptosis via interfering with glucose metabolic pathways and NAD^+^ levels [122]. In addition, PPARγ plays a pivotal role in combating oxidative stress. Zhang et al. found that the PPARγ agonist ELB00824 inhibited oxaliplatin-induced side effects—pain caused by oxidative stress and neuronal hypersensitivity. Nevertheless, it did not reduce the antitumor activity of oxaliplatin in human CRC cells in vitro [123]. We found that using the Xiao-Jian-Zhong formula in a vitro DSS-induced colitis mouse model promotes PPARγ expression by inhibiting pyroptosis and reactive oxygen species (ROS) levels. This protects intestinal mucosal integrity and prevents colitis-associated CRC [124]. Figure 3 provides a brief overview of the role of PPARγ in CRC.

## Future and challenges

The mechanisms and functions of PPARs in cancer have been extensively studied. The results of this study illustrate the potential function and influence of PPAR in CRC. These findings demonstrate the potential impact of PPARs on CRC. Still, some questions need to be considered and resolved. First, the question of how PPAR affects the growth and progression of CRC through the TME is not well understood. Second, despite a large amount of preclinical evidence, there have been no reports on the widespread and effective use of PPAR modulators in the clinic. This may be attributed to the instability of PPARs in different cell and tissue models. This makes their drug development and application a colossal challenge. Developing new PPAR agonists or antagonists and exploring their potential role in the clinical setting are necessary. Apart from that‌, is there a strong relationship between the levels of PPARs and disease progression, and can we predict prognosis and disease status based on PPAR expression? Finally, as mentioned earlier, combining PPARs and other drugs and therapies provides a valuable tool for treating CRC. Therefore, more studies and clinical trials are needed to evaluate the impact of combining PPARs with other therapies, such as immunotherapy, chemotherapy, and radiotherapy, on CRC patients’ clinical efficacy and safety.

## Conclusion

As one of the world’s deadliest malignant tumors, the exact pathogenesis of CRC remains unclear. Due to the non-specificity of early symptoms, patients are often diagnosed in advanced stages, which places a significant burden on their psychological state and physical well-being. In recent years, PPAR, a nuclear transcription factor, has been the subject of considerable research interest among the scientific community. This review summarizes the literature on PPAR and discusses the mechanisms involved in three PPARs in CRC. In the context of CRC, there is potential for designing endogenous or exogenous PPAR ligands as targeting agents. This offers new insights into the relevance of molecular targeting for cancer therapy. Therefore, future research must aim to more precisely elucidate the underlying mechanisms of CRC pathogenesis. The objective is to develop novel PPAR agonists and antagonists for more stable and practical application in clinical settings. Furthermore, the potential impact of combining PPAR modulators in patients with CRC merits investigation. PPAR modulators should be applied more widely in clinical practice, whether singly or combined. Undoubtedly, the role and targets of PPAR associated with CRC still need to be better understood, necessitating further research. We need to explore the association between CRC and PPAR more. This will facilitate the development of more effective therapeutic agents. It seems reasonable to posit that, as medical science advances, the pathophysiological mechanisms regulating PPAR in CRC will eventually be elucidated. Develop effective PPAR-targeted drugs. CRC will no longer be a global health concern.
